# The journey of breast cancer among younger women: the interplay between negative autobiographical memories and post-traumatic growth

**DOI:** 10.3389/fpsyg.2026.1788744

**Published:** 2026-03-09

**Authors:** Maria Luisa Martino, Daniela Lemmo

**Affiliations:** Department of Humanities, Federico II University, Naples, Italy

**Keywords:** autobiographical memories, breast cancer, narratives, post-traumatic growth, younger women

## Abstract

Breast cancer (BC) under 50 years is a critical, potentially traumatic experience that breaks a phase of life characterized by significant goals and expectations. Meaning-making transformation supports post-traumatic growth (PTG) that generally may occur at the end of the experience. The struggling diagnosis and treatment of BC at a young age usually remain strongly imprinted in the mind and body within the autobiographical memories (AMs). Individuals who report a tendency to draw integrative meaning or life lessons from their memories exhibit high levels of adjustment, recovery, and growth. This study aims to explore the narrative interplay between the hardest, negative AMs of BC treatment and PTG processes. A total of 10 women aged under 50 were recruited at the end of medical treatment. An *ad hoc* narrative interview was administered to explore the meaning-making of the BC experience. To pursue our aim, we focused on three narrative prompts related to negative AMs associated with BC treatment and PTG. Narratives were analyzed through Interpretative Phenomenological Analysis (IPA). The analysis shows three narrative interplay trajectories between the hardest and negative AMs related to the BC medical journey that open to specific processes of growth at the end of the treatment: *From the Death Anxiety to Self-Resignification; From Physical Suffering to the Choice of Priority; From the Temporality Collapse to the Present Moment Contact.* They focus on three distinct yet interconnected aspects of PTG: identity, relational, and temporal levels. The findings confirm how PTG is generated by women’s ability to transform the hardest and negative meaning of AMs of the experience. The presence of particularly difficult AMs attests to the psychic process associated with PTG. This process not only serves as an outcome but also facilitates meaning-making aimed at drawing integrative meaning, used for growth, either for oneself or one’s life from memory itself. Despite the limitations of this study, it highlights the supportive use of AMs in a psycho-oncology context as a device to reflect on how BC women have internalized the meanings of cancer experience. It also aims to construct a setting conducive to their transformation into a growth-oriented one.

## 1 Introduction

### 1.1 Breast cancer journey in younger women: from suffering to post-traumatic growth

The onset of breast cancer (BC) under 50 years of age is a critical, potentially traumatic experience that has broken a woman’s life, characterized by significant goals and expectations (Martino et al., 2021). The World Health Organization (WHO) estimated that there were approximately 19.3 million new cancer cases and nearly 10 million cancer deaths in 2020 (World Health Organization, 2021). The highest incidence is among individuals aged 34–49, with an 87% survival rate (Institute for Health Metrics and Evaluation, 2018).

On one side, BC during the young age, a faster and more advanced growth of the tumor condition is evident. Diagnosis, type of surgery, and type of treatment can determine physical and psychological effects on body image, fertility, early menopause, sexuality, and relationships with partners and children (Sebri et al., 2025; Black et al., 2020; Bolton and Isaacs, 2018; Helms et al., 2008; Peate et al., 2009). Studies show that the risk of major traumatic outcomes is a more difficult psychological adjustment undermined by fear of recurrence and anticipatory mourning (Martino et al., 2022; Jones et al., 2014). On the other side, young women are more flexible, living personal growth during cancer, transforming negative emotions into strengths, and modifying their life priorities (Martino et al., 2021).

Within a narrative socio-constructivist perspective, BC precipitates a crisis that disrupts the basic elements regulating the relationship between the internal and external worlds (Martino et al., 2015; Janoff-Bulman, 2004; Joseph and Linley, 2005), thereby interrupting continuity over time. The crisis impacts the meaning-making processes that support their personal life story and continuity of life (Brockmeier, 2000; Hjelmblink and Holmstrom, 2006; Kowalska et al., 2021; Neimeyer, 2006). This experience imposes narrative urgency on the mind, activating the need to synthesize new meanings and promoting the organization and connection of different elements of the experience (Freda and Martino, 2015). Narration can create order among events to construct sense and meaning (Bruner, 1991) of one’s experience, outlining interpretative and prefigurative coordinates of events, actions, and situations, and constructing forms of knowledge that orient the subject to action. Narration aims to support adjustment, integrate the event, and construct resources, well-being, and active coping strategies (Angus and McLeod, 2004; Hermans and Dimaggio, 2004; Neimeyer, 2004, 2006).

In addition, meaning-making transformation mediated by narrative supports post-traumatic growth (PTG) that generally may occur at the end of a critical or traumatic experience. Studies in cancer research suggest that growth involves increased spiritual development, changes in life meaning, greater valuing of life, increased personal strength, and improved relationships with others. New relationship with the body, a new spirituality, a sense of altruism, and empathy with people challenged (Calhoun and Tedeschi, 2014; Tedeschi and Moore, 2021; Jin et al., 2014; Su and Chen, 2015; Taku et al., 2015; Zhai et al., 2024). Positive, meaningful transformation following a cancer experience can sometimes also increase people’s ability to recover from the BC experience, although it is well known that negative changes must also be considered (Berktaş and Kabukcuoğlu, 2024). A comparison between cancer survivors and a selected sample of healthy people highlights that the former show a general growth against the latter in many aspects (Tomich et al., 2005).

PTG represents both the process and its outcome, showing how to go through the trauma, that is meaning-processing and transformation, and in the same time the outcome of this process (da Martins Silva et al., 2011; Barakat et al., 2006); therefore, PTG in cancer context is the positive resolution of the experience based on two psychological processes: recognition of the negative effect of the event, and the analysis of its meaning and the possible changes of the self; the construction of a transformation for the personal life story (Brooks and Turner, 2026; Tedeschi and Moore, 2021; Almeida et al., 2022). The link between the transformation of meaning in life and post-traumatic growth in cancer patients is shown (Almeida et al., 2022). In cancer patients, PTG also plays a preventive role, supporting better quality of life (Liu et al., 2020; Kim and Son, 2021) and reducing depression and anxiety (Thakur et al., 2022).

### 1.2 The key role of autobiographical memories in breast cancer experience

Within a Narrative Identity framework (Blagov et al., 2022), autobiographical memory (AM) plays a central role in reconstructing of the personal past, connecting to the present, and prefiguring the future within an evolving self-story (Martino et al., 2024). This is a meaning-making narrative of the self that serves to guarantee a sense of unity, coherence, and purpose in one’s own life (McAdams, 2008; Freda et al., 2023). Between memories and the narratives of memories, there is a circular and dynamic relationship of transformation of meanings over time (Nelson and Fivush, 2004) through the re-emergence of autobiographical material and its structuring into a narrative; subjects can benefit from cognitive and affective information but also convert their memories into learning opportunities and meaning for life (Blagov and Singer, 2004; Singer and Blagov, 2004). Studies strongly suggest that autobiographical narratives play a key role in successful problem solving, coping processes, and the pursuit of personal goals and growth (Nelson and Fivush, 2004; Fivush et al., 2011). Furthermore, optimal and redemptive integration of a negative life experience into the self is an outcome observed among post-cancer patients with good adaptation (Martínez-Hernández and Ricarte, 2019) and a more general indicator of psychological adjustment (Blagov et al., 2022). The struggling diagnosis of breast cancer and related treatments during a young age usually remain strongly imprinted in the mind and body within the AM of women (Sebri et al., 2025). Individuals who show a strong tendency to derive integrative meaning or life lessons from their memories report high levels of adjustment, recovery, and growth following emotionally challenging experiences (Singer and Blagov, 2004; Blagov and Singer, 2004; Singer and Conway, 2011; Blagov et al., 2022). Meaning is considered a key element for good mental health and psychological well-being (Fischer et al., 2021). To date, few studies have examined the interplay between the hardest negative narrative AMs of BC medical literature and processes of PTG at the end of BC treatment. The aim of this study is to fill this gap, highlighting this interplay, starting from the autobiographical narratives of younger women who have finished treatments for breast cancer. Yet these memories could be a valuable source of self-exploration and guidance in efforts to construct resilience, PTG, and integration in cancer survivors (Nieto et al., 2019).

## 2 Materials and methods

### 2.1 Institutional review board statement

This study was approved by the Ethical Committee of the National Cancer Institute Pascale of Naples under protocol number 36 of 18 January 2018. The study adhered to the American Psychological Association’s Ethical Principles and Code of Conduct and the principles of the Declaration of Helsinki. The participants signed a written informed consent about the study’s aims and procedures and were assured that their participation was voluntary and that their responses would remain anonymous. The study was conducted in collaboration with the hospital’s psycho-oncology unit.

### 2.2 Participants

The women who took part in this research were identified from medical reports in collaboration with medical staff and the psycho-oncology unit of the hospital according to the following criteria:

Eligibility criteria: First access to the hospital before the age of 50; diagnosis of infiltrating ductal BC.

Exclusion criteria: metastatic disease (stage IV); neoadjuvant therapy; psychotherapeutic treatments in progress.

The women who met our eligibility criteria were contacted by phone and invited to participate in the 1-day meeting. This was to explain in detail the research aim of the psychologists, researchers, and the psychologists of the hospital’s psycho-oncology unit.

At the onset of diagnosis communication, 50 women were recruited during the follow-up phase of medical treatment (1 year after the diagnosis) at the end of medical treatment, 10 women participated in the narrative interview. Women were younger than 50 years (M = 44.4; standard deviation [SD] = 4.5). The meeting took place in an *ad hoc*, quiet room in the hospital.

### 2.3 Tools

In an *ad hoc* narrative interview to explore the narrative meaning-making of the breast cancer experience at a young age in this study, we focused on three specific narrative prompts to pursue our aim.

*Could you please tell me if there has been anything or a moment that has been hardest (negative) for you during your entire treatment journey so far?*

*Do you feel like your cancer journey has changed your view of life?*

*Is there anything you feel you can draw from going through this experience, something like a legacy?*

The whole narrative interview averaged approximately 45 min and was recorded and then transcribed verbatim. The interview was conducted by two women psychologists who are experts.

in clinical psychology and narrative methodology. Same-gender membership has been represented a key factor in promoting the narrative of women. This allowed them to construct an empathic and exchange relationship with the patients.

### 2.4 Method of data analysis

We performed an Interpretative Phenomenological Analysis (IPA) (Smith et al., 2009; Smith and Fieldsend, 2021) aimed at exploring how people make sense of their experiences to deepen the understanding of a particular phenomenon, that is, the interplay between hardest negative AMs related to medical iter and processes of growth at the end of BC treatment.

The interviews were transcribed verbatim and then read and re-read to immerse in the data. The flow of data analysis was (a) movement from what is unique to a participant to what is shared among the participants; (b) description of the experience, which moves to an interpretation of the experience; (c) commitment to understanding the participant’s perspective; and (d) psychological focus on personal meaning-making within a particular context (Smith et al., 2009; Smith and Fieldsend, 2021).

Following the IPA process, initial notes, which include descriptive, linguistic, and conceptual comments, were created. The purpose of descriptive comments is to describe the content of the data. At the next level of analysis, the researchers focused on how the transcript reflected the ways in which the content and meaning were presented linguistically, and the researchers’ notes took the form of linguistic comments. In the third level of analysis, the researchers centered on a more interpretive phase by offering conceptual comments. This stage of analysis involves developing questions about meaning. At this phase, researchers begin to develop insights into the data that will allow them to develop themes in the next stage of analysis. In making conceptual comments, the researchers noted preliminary concepts regarding the participants’ overarching understanding of learning qualitative research. After completing initial note-taking on each participant’s responses, the researcher searched for emerging themes across all participants by examining discrete sections of the transcripts and simultaneously recalling what they had learned during their analysis. The themes are the product of the thoughts of participants, but also the researchers’ interpretations.

## 3 Results

The analysis shows specific narrative interplays and trajectories among the hardest negative AMs related to the BC medical journey that opens to processes of growth at the end of the treatment experience (Table 1).

**Table 1:** Narrative interplays trajectories between hardest AMs of BC and processes of PTG.

The transformative process	First trajectory narrative interplay	Second trajectory narrative interplay	Third trajectory narrative interplay
Hardest AMs of BC journey	The death anxiety	Physical suffering	The temporality collapse
	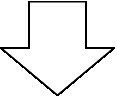	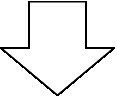	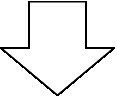
PTG	The self-resignification	The choice of priority	The present moment contact

### 3.1 First trajectory interplay: from the death anxiety to self-resignification

*Hardest memory of BC journey*: The memories are focused on the initial moments of the journey: the preoperative anxiety and the way in which the diagnosis communication was delivered by physicians. The anxiety is linked to the unknown and the uncertainty that characterize these moments, and the women symbolize them with a real risk of death. The critical moment is experienced as a biological and identity threat that undermines the role of mother and one’s existence in the world.

*“The hardest moment was before the surgery… I was terrified because I would not be able to get over it… You know, you always have that thought, ‘But… what if I fall asleep and never wake up? A. will then wait for my call.’” (ID 2).*

*“I had a doctor who gave me the news in a very, very harsh way… he said to me, ‘Did you know it was malignant?’ Exactly… it shocked me… on a human, animal level.” (ID 3).*

*Post-traumatic growth*: Life is no longer an automatic duty toward the outside world. It’s no longer something to be taken for granted, nor something to be lived to accomplish the needs and desires of others, but a precious value to be protected for oneself. We observe a shift from impulsiveness to profound reflection and awareness. The acquisition of life involves the re-signification of the self as an embodied self, no longer linked to impulsive or instinctive responses but to thoughtful reflection before acting on one’s own behalf. A new self-boundary and a greater confidence in one’s own resources appear to have been acquired by posing the embodied self as a perspective.

*“I used to be more impulsive; now I think and reflect. I’m quieter… I must be careful of myself; I must take better care of myself. That’s all… it’s not a duty, you know; maybe it should be a right, my right.” (ID 15).*

### 3.2 Second trajectory: from physical suffering to the choice of priority

*Hardest memory of BC journey*: The memories are focused on the physical suffering and pain, eminently connected to the chemotherapy phase and side effects. Physical pain and the efforts to cope with it in the memories were joined with the losses of important relationships and social support. The physical pain became unique with the psychological one, leading to disappointment, isolation, and anger.

*“The last chemo was incredibly difficult… physical pain for 13 days straight, night and day, never resting… always in pain.” (ID 1).*

*“Some people have truly disappeared… longtime friends… Now I welcome some, and I no longer welcome others. I want to be welcomed.” (ID 15).*

*Post-traumatic growth*: Life is interpreted through the lens of essentiality and truth. It has become a space that centralizes new priorities on the real aspects of the external and internal world, eliminates fiction and hypocrisy, and invests only in what is “true.” The importance of jobs and the judgment of others loses value and importance.

*“If you stop for a moment to see how many trivial things we have given importance to, you kill yourself with work all day… and then when you realize that a blow like this is coming your way, you have to stop.” (ID 1).*

*“You take away many people; you eliminate them, relatives, you eliminate them… you avoid hypocrisy… you must not waste time, neither with people nor with things.” (ID 3).*

### 3.3 Third trajectory: from the temporality collapse to the present moment contact

*Hardest memory of BC journey*: The memories center on waiting times at the hospital and the loss of personal control over the subjective time of one’s own life due to system inefficiencies, prolonged waits, and uncertain diagnoses. This appears to be a condition of liminality in which the women assume the role of the sick person.

*“Seven months have passed since they told me I needed to have another operation… they made me redo my medical records three times. It was something I could not stand… it completely derailed my thinking.” (ID 29).*

*Post-traumatic growth*: A new interpretation of life emerged based on the contact with day-by-day moments. Since the future is uncertain, women learn to be grateful for the contact with the present. Anxiety about the future decreases as life is marked by uncontrollable events. A strength based on the knowledge of having overcome a major adversity emerged.

*“I think life should be lived day by day… not to think too much about the future… start by being thankful for each day that is there.” (ID 29).*

*“Do not get discouraged; most of the bad things in life can be faced, and you can get back to living… I’ve definitely hardened myself, yes.” (ID 50).*

## 4 Discussion

The analyses conducted and the narrative interplay trajectories confirm how PTG is generated by the subjects’ ability to transform the meaning of the hardest and negative AMs related to a critical, potentially traumatic experience. This includes breast cancer at a young age (Brooks and Turner, 2026; Tedeschi and Moore, 2021; Almeida et al., 2022). The presence of particularly difficult and negative memories related to the cancer treatment journey attests to the psychic process associated with PTG. Not only as an outcome but also as a process of meaning-making aimed at drawing an integrative meaning for oneself or one’s life from the memory itself. This is to enable its reclamation and use for personal growth (Martino et al., 2023). The transition from negative memories to the ability to draw meaning from them and from the entire experience will pave the way for processing the illness experience; beings seem to arise from the transformations of the underlying meanings condensed in negative memories through a redemptive transformation of those meanings (McAdams, 2008). The three interplays and trajectories emerged to focus on three distinct yet interconnected aspects of PTG: the identity level, the relational level, and the temporal level.

In the first narrative interplay trajectory, the salience of the hardest memories on death anxiety shows the centrality of the assault on the body and its survival. Through the communication of the diagnosis or preoperative intervention, it directs post-traumatic growth processes toward a greater centering of the embodied self. With its needs and desires (Durt, 2020; Sebri et al., 2020). This self is re-signified and transformed by establishing boundaries and choices and recalibrating the internal relationship between emotions and thoughts in life.

In the second narrative trajectory, the salience of hard memories focuses on physical suffering connected to the most difficult moments of the treatment process, such as chemotherapy. The loss of body parts (such as hair or breast tissue) merges with the loss of supportive relational aspects, making women feel alone and abandoned. These experiences guide post-traumatic growth toward prioritizing one’s relationship with one’s own life, enabling one to reach the essential and true aspects of relationships with others by eliminating pretense and hypocrisy. They also help one surround oneself with real people one can count on for the future. Truth serves as a compass for one’s life (Wang et al., 2024; Cao et al., 2018).

In the third narrative interplay trajectory, the salience of the hardest memories centers on a collapse of temporality, wherein the woman loses control over her subjective time in her relationship with the illness and the hospital. She loses control of herself and her future life. The collapse and the suspension of subjective temporality, such as a liminal condition (Little et al., 2022). The expropriation of one’s own time and uncertain expectations gives direction to post-traumatic growth processes toward the possibility of experiencing contact with the present moment in one’s life. This reduces control over future events and savors mindful aspects of the relationship with life and its present moments (Karekla et al., 2022).

### 4.1 Clinical implication, limitation, and conclusion

Despite the limitations of this study, including a small sample size, the context-dependent findings, and the absence of external quantitative measures. It highlights the interplay between narrative meaning and the transformation of the most difficult and negative autobiographical memories among younger women. It also highlights processes of post-traumatic growth and recovery at the end of the medical journey. In particular, the use of autobiographical narrative memories in a psycho-oncology context can offer insight into how BC women have internalized past events regarding the cancer treatment. This internalization reflects their sense of bodily integrity, psychological health, and their relationship with health providers (Martino et al., 2023).

The use of autobiographical memory narratives, in different formats and settings, is helpful not only during survivorship but also during earlier phases of medical care to address the discontinuities in her narrative caused by BC. This helps to reconstruct a sense of coherence and sustained meaning. This is despite an altered sense of self (Martino et al., 2025).

As previous studies suggest, particularly for younger women, it becomes central to provide meaning-centered supportive interventions that can potentiate a positive adjustment and possibly growth from the cancer experience to pursue life achievements after BC. The ability to detect women struggling with meaning can be the key to assisting these patients’ adjustment to cancer by suggesting psychological support or specialized meaning-centered interventions (Almeida et al., 2022).

In addition, our findings underscore the importance of the linguistic, narrative, and thematic approaches for a more in-depth understanding of PTG and its relationship with other processes, thereby informing the tailoring of supportive interventions (Scrignaro et al., 2018). Further studies should deepen this interplay across the different phases of treatment and cancer types.

## Data Availability

The raw data supporting the conclusions of this article will be made available by the authors, without undue reservation.
